# Cardiopulmonary bypass complications and their predictors in open-heart surgery at a tertiary cardiac care facility in Tanzania

**DOI:** 10.21542/gcsp.2026.11

**Published:** 2026-04-30

**Authors:** Alfred D. Luvakule, Silas F. Gamba, Edwin R. Lugazia, Alex Loth

**Affiliations:** 1 Department of Anesthesiology, School of Medicine, Muhimbili University of Health & Allied Sciences, Dar es Salaam, Tanzania; 2 Department of Cardiology and Cardiothoracic Surgery, Benjamin Mkapa Hospital, Dodoma, Tanzania; 3 Department of Anesthesiology, Jakaya Kikwete Cardiac Institute, Dar es Salaam, Tanzania

## Abstract

**Background**: Cardiopulmonary bypass (CPB) is integral to open-heart surgery but carries significant risks of systemic inflammatory response and organ-specific complications. In low-resource settings, limited local data can impede targeted prevention. This study assessed the frequency and predictors of CPB-related complications at the Jakaya Kikwete Cardiac Institute, Tanzania.

**Methods**: A retrospective review examined 195 pediatric and adult patients who underwent open-heart surgery in 2020. Data from perfusion records and ICU charts were analyzed using descriptive statistics and univariate logistic regression.

**Results**: Of the cohort, 55.4% were male and 52.3% were under 16 years. Valvular heart disease was the most common diagnosis (42.1%). At least one major complication occurred in 31.3% of patients, with cardiovascular (10.8%) and bleeding (9.2%) events predominating. Prolonged ICU stay affected 52.8% of cases and in-hospital mortality was 4.1%. Key predictors of complications included younger age, diagnosis type, procedure type, CPB duration, and cross-clamp time. CPB time exceeding 120 min significantly increased the likelihood of prolonged ICU stay.

**Conclusions**: CPB-related complications represent a substantial burden in this setting. Minimising CPB and cross-clamp durations may improve outcomes. Prospective locally driven research is needed to refine management strategies.

## Introduction

Cardiopulmonary bypass (CPB) is an essential technique in open-heart surgery, enabling a bloodless, motionless operative field by diverting blood from the heart and lungs^1^. The process involves arresting the heart with cardioplegia while an extracorporeal circuit maintains circulation and oxygenation^1–3^. Although CPB is vital for many complex cardiac procedures, it is associated with important postoperative complications, including systemic inflammatory response syndrome, cardiovascular instability and multi-organ dysfunction^4^. These complications arise from factors such as contact between blood and the bypass circuit’s artificial surfaces, aortic cross-clamping, cardioplegia, transfusion of allogeneic blood and operative trauma^5,6^. Inflammatory and embolic mechanisms can impair the function of vital organs, particularly the heart, brain, lungs and kidneys, leading to increased morbidity and mortality when physiological reserves are exceeded^4,7^.

Globally, more than two million CPB procedures are performed each year, with over 250,000 in the United States alone^8,9^. In contrast, sub-Saharan Africa (SSA) faces significant challenges due to limited resources, optimized personnel and infrastructure, resulting in an estimated open-heart surgery rate of only two per million population^8^. In Tanzania, access to advanced CPB technology, such as biocompatible circuit coatings, improved oxygenators and modern antifibrinolytic drugs, is inconsistent. Prolonged CPB times, suboptimal postoperative monitoring and the absence of optimized perioperative protocols may increase the risk of adverse outcomes^6,10^.

Most research into CPB-related complications originates from high-income countries, where clinical environments differ markedly from those in SSA. Differences in surgical case mix, patient characteristics, disease stage at presentation and perioperative support limit the applicability of international data to low-resource settings^11^. To date, no published study has evaluated the predictors of CPB-related complications in Tanzania, and few have done so within the broader SSA context. This knowledge gap makes it difficult to develop evidence-based strategies for improving outcomes.

This study aimed to determine the incidence and predictors of CPB-related complications in patients undergoing open-heart surgery at the Jakaya Kikwete Cardiac Institute, Tanzania’s national cardiac referral centre. By identifying key risk factors associated with adverse outcomes, such as prolonged intensive care unit stays and postoperative organ dysfunction, we sought to provide evidence that could guide preventive strategies, optimize resource allocation and align local surgical outcomes with global standards.

## Methods

This retrospective chart review was conducted at the Jakaya Kikwete Cardiac Institute (JKCI), Tanzania’s national, university-affiliated tertiary cardiac referral centre. This study was approved by the Muhimbili University of Health and Allied Sciences Research Ethics Committee and conducted in accordance with the Declaration of Helsinki. As this was a retrospective chart review, the requirement for written informed patient consent was waived by the committee.

**Figure 1.**
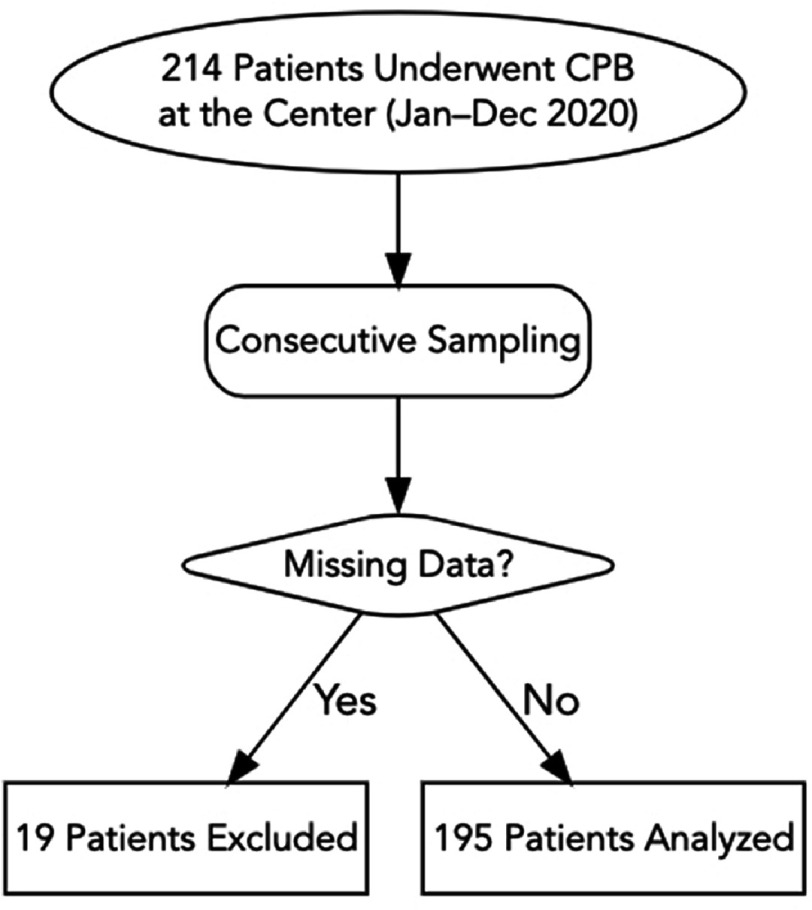
Flowchart for patient selection, exclusions, and final sample.

We included all patients who underwent cardiac surgery requiring cardiopulmonary bypass between 1 January and 31 December 2020. We identified 214 patients during this period. Of these, 19 (8.9%) were excluded due to incomplete or missing medical records: 11 were missing critical intraoperative perfusion data (CPB duration or cross-clamp time), 5 had incomplete postoperative ICU charts, and 3 had missing preoperative diagnostic information. Excluded patients did not differ systematically from included patients with respect to age or sex distributions based on available administrative data, although formal comparison was limited by the incomplete records. The final analytical sample comprised 195 patients (Figure 1). Sampling was consecutive and non-probabilistic.

Data were obtained from perfusion records, intensive care unit charts and operative notes, and recorded using a structured data collection form. Variables included demographic characteristics (age, sex), preoperative diagnoses classified according to American College of Cardiology/American Heart Association guidelines, and intraoperative details (type of surgery, duration of CPB, aortic cross-clamp time). Postoperative data included major complications, duration of intensive care unit stay, and mortality. Data collection was performed by trained intensive care nurses and perfusionists, following a predefined protocol to ensure accuracy.

### Operational definition of ‘complication’

Postoperative complications were defined and classified as follows, consistent with established criteria where referenced.

**Cardiovascular complications** were defined as any of the following occurring within 30 days of surgery:

• **Haemodynamic instability:** sustained systolic blood pressure below 90 mmHg or above 160 mmHg requiring intervention
• **Low cardiac output syndrome:** cardiac index below 2.0 L/min/m^2^ with end-organ hypoperfusion necessitating inotropic or mechanical circulatory support
• **Unresponsive bradycardia**
• **Newly identified postoperative arrhythmia**, including atrial fibrillation and sustained tachyarrhythmias, requiring pharmacological or electrical treatment

**Bleeding complications** were defined as any clinically significant haemorrhage meeting at least one of the following criteria:

• Re-exploration for bleeding
• Transfusion of four or more units of packed red blood cells within 24 h
• A haemoglobin fall exceeding 4 g/dL not attributable to haemodilution

**Pulmonary complications** were defined as any of the following:

• Radiographically confirmed atelectasis with clinical significance
• Pulmonary oedema requiring diuresis or ventilatory support
• Pleural effusion requiring intervention

**Renal complications** were defined as acute kidney injury (AKI) according to KDIGO criteria, comprising any of the following:

• Serum creatinine increase of ≥0.3 mg/dL within 48 h
• Serum creatinine rise to ≥1.5 × baseline within 7 days
• Urine output below 0.5 mL/kg/hour for six or more consecutive hours

**Metabolic complications** were defined as any of the following:

• Clinically relevant metabolic acidosis (pH below 7.30 or base excess below −6)
• Significant hyperkalaemia (serum potassium above 6.0 mmol/L)
• Persistent hyperglycaemia (blood glucose above 10 mmol/L) requiring insulin infusion

**Infectious complications** were defined as any postoperative infection occurring during the ICU stay, including:

• Surgical site infection
• Pneumonia
• Bloodstream infection

Diagnosis required confirmation by microbiological culture or established clinical criteria.

**Prolonged ICU stay** was defined as an ICU admission exceeding 48 h, consistent with prior studies in cardiac surgery.

### Data analysis

Analyses were conducted using SPSS Statistics version 23 (IBM Corp., Armonk, NY, USA). Continuous variables were expressed as mean (SD), or median (IQR) where data were non-normally distributed; categorical variables were expressed as frequency and percentage.

The *χ*^2^ test was used to assess univariate associations between categorical variables and the occurrence of postoperative complications. Univariate binary logistic regression was then applied to estimate unadjusted odds ratios (ORs) with 95% confidence intervals (CIs) for potential predictors. A formal multivariable model was not fitted, as the number of outcome events (*n* = 61) was insufficient relative to the number of candidate predictor variables; doing so would have risked overfitting. All reported ORs and CIs therefore derive from univariate analyses, which represents a key limitation of the study. Statistical significance was set at *p* < 0.05.

## Results

### Demographic and preoperative characteristics

Of 195 patients, 108 (55.4%) were male. The largest age group was those younger than 16 years (102; 52.3%), while 42 (21.5%) were older than 45 years. Nearly half (94; 48.2%) weighed <30 kg, and 47 (24.1%) had a body surface area ≤0.6 m^2^. Repeat surgery was uncommon (6; 3.1%). Preoperative anaemia was present in 72 (36.9%) patients with haemoglobin <10 g/dl (Table 1).

**Table 1:** Baseline demographic and preoperative characteristics of the patients open heart surgery. Hgb, haemoglobin. All haemoglobin values are expressed in g/dL.

**Variable**	**Category**	**Frequency, (*N* = 195)**	**Percentage (%)**
**Sex**	Female	87	44.6
	Male	108	55.4
**Age (years)**	0–15	102	52.3
	15–30	26	13.3
	30–45	25	12.8
	>45	42	21.5
**Weight (Kilograms)**	0–30	94	48.2
	31–60	46	23.6
	61–90	45	23.1
	>90	10	5.1
**Body surface area (M** ^**2**^ **)**	0-0.6	47	24.1
	0.6–1.1	44	22.6
	1.1–1.7	56	28.7
	>1.7	48	24.6
**Redo operation**	Yes	6	96.9
	No	189	3.1
**Pre-operative Hgb (g/dl)**	<6.5	9	4.6
	6.5–8.0	15	7.7
	8.0–10	48	24.6
	10.0–12	51	26.2
	12.0–18	64	32.8
	>18	8	4.1

### Diagnostic categories and surgical procedures

Valvular heart disease was the most frequent diagnosis (82; 42.1%), followed by acyanotic congenital anomalies (51; 26.2%) and cyanotic congenital anomalies (33; 17.4%). Valve replacement was the most common procedure (73; 37.4%), followed by patch closure of septal defects (41; 21.0%) and repair of tetralogy of Fallot (27; 13.8%). Coronary artery bypass grafting was performed in 22 patients (11.3%) (Table 2).

**Table 2:** Type and frequency of diagnoses and surgical procedures among patients who underwent open heart surgery. Reconstruction surgery = aortic arch reconstruction; combined = patch closure + valve, patch closure+ PDA ligation, Glenn hunt + PDA ligation, valve replacement + annuloplasty; palliative surgery = Glenn shunt, BT shunt; valve replacement= MVR, DVR, AVR, Valve repair = Mv Repair; acyanotic anomalies = VSD, ASD, PDA, AVSD, VSD + PDA, partial Av canal, coarctation of aorta; cyanotic anomalies = TOF, TAPVR, tricuspid atresia (TA), pulmonary atresia, double outlet right ventricle; coronary artery disease = CAD, TVD, DSS; valvular heart disease = mitral valve regurgitation (MR), mitral stenosis (MS), aortic regurgitation, aortic stenosis (AS), tricuspid regurgitation (TR), MR + TR, MS + AS; acyanotic and valvular heart diseases = MS + MR + PDA, VSD + AS, ASD + PDA + TR; cyanotic and valvular heart diseases = TA + ASD.

**Variable**	**Frequency (*N* = 195)**	**Percentage (%)**
**Diagnosis**		
Acyanotic and valvular heart diseases	5	2.6
Acyanotic heart anomalies	51	26.2
Coronary heart disease	22	11.3
Cyanotic and valvular heart diseases	1	0.5
Cyanotic heart anomalies	33	17.4
Valvular heart diseases	82	42.1
**Procedure**		
Reconstruction surgery	1	0.5
Coronary artery bypass graft	22	11.3
Combined procedure	18	9.2
Palliative surgery	7	3.6
Patch closure	41	21
PDA ligation	2	1
TAPVR correction	1	0.5
ToF repair	27	13.8
Valve repair	3	1.5
Valve replacement	73	37.4

### Complication profile and outcomes

Perioperative complications occurred in 61 patients (31.3%), most frequently cardiovascular events (21; 10.8%), bleeding (18; 9.2%), and pulmonary complications (12; 6.2%). Metabolic, infectious, and renal complications each occurred in ≤3.1% (Table 3). Prolonged ICU stay (>2 days) was observed in 103 patients (52.8%). There were 8 in-hospital deaths (4.1%), all occurring within the ICU during the postoperative period; 6 of these deaths were preceded by a documented major complication (4 cardiovascular, 1 bleeding, 1 combined metabolic and renal). The remaining 2 deaths occurred in patients without a recorded major complication from the pre-specified categories, and were attributed to refractory low cardiac output in the early postoperative period. Cause-specific mortality data were unavailable in full for all cases due to the retrospective design.

**Table 3:** Cardiopulmonary complications, length of ICU stay and outcome of patients undergoing open heart surgery. Cardiovascular complications = hypertension, hypotension, low cardiac output syndrome, bradycardia, unresponsive bradycardia, postoperative arrhythmias (atrial fibrillation, tachyarrhythmia); Metabolic abnormalities = acidosis, hyperkalemia, hyperglycemia; Pulmonary complications = atelectasis, pulmonary oedema, pleural effusion; Renal complications = acute kidney injury

Variable	Frequency (*N* = 195)	Percentage (%)
Complications		
Yes	61	31.3
No	134	68.7
Type of complications		
Metabolic	6	3.1
Cardiovascular	21	10.8
Bleeding	18	9.2
Infection	6	3.1
Pulmonary	12	6.2
Renal	2	1
Post-operative arrhythmias	8	4.1
Length of ICU stay		
≤2 days	92	47.2
>2 days	103	52.8
Outcome		
Alive	187	95.9
In-hospital Death	8	4.1

**Figure 2.**
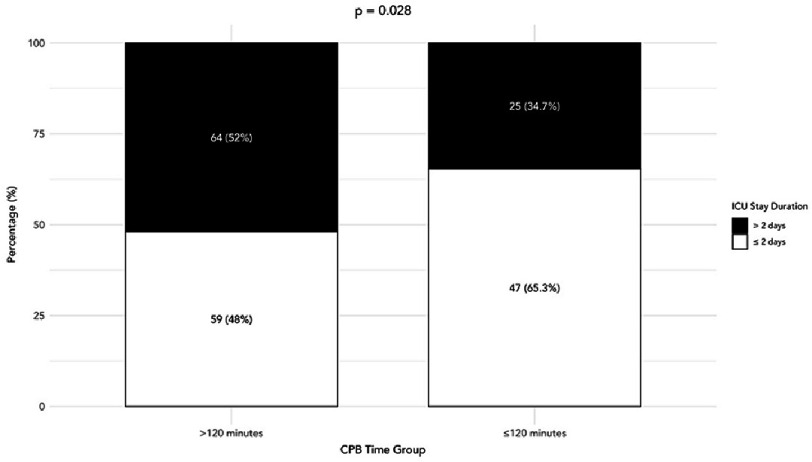
Distribution of prolonged and non-prolonged ICU stay by CPB time group.

Prolonged ICU stay (>2 days) was observed in 103 (52.8%). There were 8 in-hospital deaths (4.1%). Patients with cardiopulmonary bypass (CPB) times >120 min were more likely to have prolonged ICU stays than those with shorter durations (64/123 [52.3%] vs 25/72 [35.1%], *p* = 0.049; Figure 2).

### Factors associated with complications

Unadjusted associations between selected variables and the occurrence of any postoperative complication are presented in Table 4. Complication rates differed significantly by age group (*p* = 0.008), with the highest proportions in patients aged 0–16 years (OR 2.41, 95% CI [1.14–5.10] vs >45 years) and 17-30 years (OR 4.54, 95% CI [1.32–15.64]). Sex was not significantly associated with complications (OR 1.37; 95% CI [0.74–2.53]; *p* = 0.95). Diagnosis type was significantly associated (*p* = 0.015), with the highest unadjusted OR in coronary artery disease (OR 5.92, 95% CI [1.98–17.67] vs acyanotic anomalies). Procedure type was also associated (p = 0.037); CABG (OR 7.22, 95% CI [2.25–23.1]) and palliative surgery (OR 6.67, 95% CI [1.22–36.7]) carried the highest unadjusted odds relative to patch closure.

**Table 4:** Univariate associations (unadjusted odds ratio) between potential predictors and cardiopulmonary complications and selected variables among patients who underwent open heart surgery.

Variable	Category	Complications Yes n (%) No n (%)		OR (95% CI)	*p*value
Sex	Female	24 (39.3%)	63 (47.0%)	1.37 (0.74 –2.53)	0.947
	Male	37 (60.7%)	71 (53.0%)	—(Reference)	
Age (years)	0–16	26 (42.6%)	76 (56.7%)	2.41 (1.14 –5.10)	**0.008**
	17–30	4 (6.6%)	22 (16.4%)	4.54 (1.32 –15.64)	
	31–45	12 (19.7%)	13 (9.7%)	0.90 (0.33 –2.41)	
	>45	19 (31.1%)	23 (17.2%)	—	
Transfusion	Yes	78 (64.5%)	43 (35.5%)	1.72 (0.90 –3.25)	0.101
	No	56 (75.7%)	18 (24.3%)	—	
Diagnosis	Acyanotic & valvular	3 (60.0%)	2 (40.0%)	2.73 (0.40 –18.55)	**0.015**
	Acyanotic anomalies	41 (80.4%)	10 (19.6%)	—	
	Coronary heart disease	9 (40.9%)	13 (59.1%)	5.92 (1.98 –17.67)	
	Cyanotic & valvular	0 (0%)	1 (100%)	NA	
	Cyanotic anomalies	21 (61.8%)	13 (38.2%)	2.54 (0.95 –6.75)	
	Valvular heart disease	58 (70.7%)	24 (29.3%)	1.70 (0.73 –3.94)	
Procedure	Reconstruction surgery	0 (0%)	1 (100%)	NA	**0.037**
	CABG	9 (40.9%)	13 (59.1%)	7.22 (2.25 –23.1)	
	Combined	12 (66.7%)	6 (33.3%)	2.50 (0.70 –8.93)	
	Palliative surgery	3 (42.9%)	4 (57.1%)	6.67 (1.22 –36.7)	
	Patch closure	35 (83.3%)	7 (16.7%)	—	
	PDA ligation	2 (100%)	0 (0%)	NA	
	TAPVR correction	1 (100%)	0 (0%)	NA	
CPB time (mins)	0–60	6 (54.5%)	5 (45.5%)	2.56 (0.68 –9.58)	0.009
	61–120	46 (75.4%)	15 (24.6%)	—	
	121–180	55 (77.5%)	16 (22.5%)	0.89 (0.40 –2.01)	
	>180	27 (51.9%)	25 (48.1%)	2.84 (1.27 –6.30)	
Cross-clamp time (mins)	0–60	29 (72.5%)	11 (27.5%)	—	**0.001**
	61–120	78 (78.0%)	11 (11.0%)	0.37 (0.15 –0.95)	
	121–180	21 (55.3%)	17 (44.7%)	2.13 (0.83 –5.48)	
	>180	6 (35.3%)	11 (64.7%)	4.83 (1.42 –16.3)	
ICU stay	≤2 days	78 (84.8%)	14 (15.2%)	—	**0.001**
	>2 days	56 (54.4%)	47 (45.6%)	4.67 (2.34 –9.31)	

**Notes.**

OR odds ratio CI confidence interval CABG coronary artery bypass grafting CPB cardiopulmonary bypass ICU intensive care unit ref reference category

* Statistically significant at *p* < 0.05. All ORs are unadjusted (univariate).

Longer CPB duration was linked to higher complication rates (*p* = 0.009). Compared with 61-120) 61–120 min, procedures lasting >180 min carried more than double the unadjusted odds of complication (OR 2.84, 95% CI [1.27–6.30]).Longer aortic cross-clamp times were similarly associated (*p* < 0.001), with those exceeding 180 min having an OR of 4.83 (95% CI [1.42–16.3]) compared to the <60 min reference group. Blood transfusion was associated with higher complication rates (35.5% vs 24.3%), although this did not reach statistical significance (*p* = 0.10).

Pertaining subtype of complications, cardiovascular (*p* = 0.035), bleeding (*p* = 0.048), and renal (*p* = 0.032) complications were significantly associated with longer CPB durations, whereas metabolic, infectious, pulmonary, and arrhythmic complications were not (Table 5).

**Table 5:** Associations between type of complications, outcome and CPB time among patients who underwent open heart surgery.

Complication	CPB time(minutes)				*P*-value
	**≤60 n (%)**	**61–120n (%)**	**121–180** ** n (%)**	**>180** ** n (%)**	
Metabolic abnormalities	0 (0.0%)	2 (33.3%)	0 (0.0%)	2 (33.3%)	0.924
					
Cardiovascular complications	0 (0.0%)	5 (23.8%)	5 (23.8%)	11 (52.4%)	**0.035**
					
Bleeding	2 (11.1%)	4 (22.2%)	5 (27.8%)	7 (38.9%)	**0.048**
Infection	0 (0.0%)	3 (50.0%)	1 (16.7%)	2 (33.3%)	0.613
					
Pulmonary complication	0 (0.0%)	4 (33.3%)	2 (25.0%)	5 (41.7%)	0.517
					
Renal complication	0 (0.0%)	0 (0.0%)	0 (0.0%)	2 (100%)	**0.032**
Postoperative arrhythmias	2 (25.0%)	2 (25.0%)	1 (12.5%)	3 (37.5%)	0.062

**Notes.**

CPB cardiopulmonary bypass

* Statistically significant at *p* < 0.05.

## Discussion

In this cohort from a Tanzanian tertiary-care centre, one-third of patients undergoing open-heart surgery with cardiopulmonary bypass (CPB) developed a major postoperative complication. Cardiovascular, bleeding, and pulmonary events were the most frequent. Prolonged CPB and aortic cross-clamp times, younger age, and certain diagnostic categories were significantly associated with these outcomes on univariate analysis, which in turn contributed to longer intensive care unit (ICU) stays.

The complication rate observed (31.3%) is lower than the 50–70% reported in several low- and middle-income country series, such as the 70.2% in an Indian cohort^12^ and lies within the lower range reported in contemporary high-income settings (20–40%)^1,13^. The in-hospital mortality rate (4.1%) is between the 2.2% reported in the STS Adult Cardiac Database and the 9.9% seen in a Jordanian study^14,15^. This comparatively favourable profile may reflect selective referral of less complex cases, younger patient age, and lower comorbidity burden, particularly from rheumatic heart disease^16^, as well as possible under-recognition of subclinical complications in the absence of advanced post operative diagnostic monitoring of complications.

Male sex was associated with a non-significantly higher complication risk (OR 1.37), consistent in direction with Patra et al., who reported a 1.8-fold increase in males after CPB^12^. Males may be more prone to ischaemia–reperfusion injury, coagulation abnormalities, and myocardial stress. While females have been reported to experience more systemic inflammatory responses^17^. These findings highlight the complex, multifactorial mechanisms potentially driving sex differences in postoperative outcomes, though no statistically significant association was observed in this study.

Cardiovascular complications occurred in roughly one in nine patients and were strongly linked to prolonged CPB, consistent with mechanisms involving ischaemia–reperfusion injury, systemic inflammation, and microvascular dysfunction^2,4,6^. Rates were comparable to those reported in China (13.5–42.5%) [18] and the UK (20–25%)^4^. Renal complications were rare (1%) compared with 7.8–30% reported elsewhere^18,19^, which may reflect genuine baseline differences as well as limited perioperative creatinine monitoring.

Longer CPB and cross-clamp times were associated with higher odds of cardiovascular, bleeding, and renal complications, in keeping with evidence linking durations over 180 min with multi-organ dysfunction^19–21^. Mechanistically, prolonged extracorporeal circulation may trigger inflammatory cascades, disrupts autoregulation, and increases endothelial permeability. In contrast, we found no significant association between CPB time and pulmonary or metabolic complications, diverging from previous reports^22^. This may be due to limited statistical power or differences in intraoperative ventilatory management.

More than half of the patients had an ICU stay exceeding two days, a proportion higher than the 24–26% reported elsewhere^23–25^. Prolonged ICU stays may indicate a higher complication burden in this cohort, or may reflect resource and capacity constraints that delayed safe discharge despite clinical improvement. The association between prolonged ICU stay and increased complications mirrors findings by Nissinen et al.^5^ and Salis et al.^19^, underscoring the operational impact of prolonged CPB in resource-limited settings.

Several strategies may be associated with reduced CPB-related complications. Tranexamic acid is associated with reduced bleeding but carries a dose-dependent risk of postoperative seizures, limiting its broad applicability^26^. While plasma, prothrombin complex concentrates, and bypass agents may reduce coagulopathy, robust evidence in resource-limited settings remains scarce^27,28^. Strategies such as blood conservation protocols, refined myocardial protection, and miniaturized CPB circuits show promise but demand significant investment and infrastructure^29,30^. In the present context, reducing CPB and cross-clamp times through targeted surgical training and streamlined workflows appears the most practical approach.

## Limitations

This study has several important limitations. First, the retrospective design limited the completeness of complication capture, particularly for subclinical events. Second, the number of outcome events (n = 61) was insufficient to support robust multivariable logistic regression analysis; consequently, all reported associations are unadjusted and confounding between predictor variables cannot be excluded. For example, more complex procedures tend to have longer CPB and cross-clamp times, meaning these factors are not independent in practice. Third, the small number of specific complication types (e.g., 2 renal events) limits the reliability of subgroup analyses. Fourth, absence of advanced postoperative monitoring may have led to underestimation of renal, metabolic, and neurological complications. These findings should therefore be interpreted as hypothesis-generating rather than definitively establishing independent predictors of complications.

## Conclusion

Based on the findings of this observational study, CPB and cross-clamp duration were associated with higher rates of postoperative complications and prolonged ICU stay. Efforts to reduce these durations and to optimise perioperative care may contribute to improved outcomes, and are particularly relevant in resource-limited settings where prolonged recovery affects institutional capacity. These findings should be interpreted within the context of a univariate, retrospective analysis. Further prospective multicenter studies with standardized protocols and registries are needed to improve benchmarking, guide quality improvement, and support resource allocation tailored to this context.

## Funding Sources

No external funding was received

## Declaration Of Competing Interests

There are no conflicts of interest to disclose.

## Author Contributions

A.D.L.: Conceptualization, Methodology; Data Curation, Writing- Original draft preparation

E.R.L.: Methodology, Supervision, Writing- Reviewing and Editing.

S.F.G.: Conceptualization, Investigation, Writing- Reviewing and Editing.

## Data Availability Statement

All data requests should be submitted to the corresponding author for consideration; access to anonymised data may be granted following review.

## Acknowledgements

We are indebted to the surgical, anaesthesia, and ICU teams at Jakaya Kikwete Cardiac Institute and the Institute’s management for their permission and support.
